# Persistent Genital Arousal Associated With a Tarlov Cyst: A Case Report

**DOI:** 10.7759/cureus.96485

**Published:** 2025-11-10

**Authors:** Arhum Mustajab, Eleanor Barnett

**Affiliations:** 1 Obstetrics and Gynaecology, University Hospitals of Derby and Burton NHS Foundation Trust, Derby, GBR

**Keywords:** case report, perineural cyst, persistent genital arousal, sacral nerve root, tarlov cyst

## Abstract

Persistent genital arousal syndrome (PGAS) is a rare and distressing condition characterised by spontaneous genital arousal in the absence of sexual desire or stimulation. Although its aetiology is multifactorial, neurogenic causes such as Tarlov cysts are increasingly recognised.

We report the case of a 79-year-old woman who presented with persistent, intrusive genital arousal unrelieved by orgasm, developing shortly after initiating vaginal oestrogen therapy. Despite discontinuation, her symptoms persisted and caused significant psychological distress. Gynaecological examination was unremarkable, prompting consideration of a neurogenic cause. MRI revealed small Tarlov cysts at the S2 level and filum terminale, consistent with possible sacral nerve root irritation. The patient was referred to neurosurgery and remains under multidisciplinary conservative management, given her significant comorbidities.

This case highlights the importance of recognising lumbosacral pathology as a potential cause of PGAS, particularly when other distinguishing features are absent or symptoms are refractory. Early neuroimaging in unexplained cases may reduce diagnostic delay and improve outcomes through timely, individualised, and multidisciplinary care.

## Introduction

Persistent genital arousal syndrome (PGAS), also known as persistent genital arousal disorder, is a rare and distressing condition affecting approximately 1%-4% of the general population [1,2]. It is characterised by involuntary and persistent genital arousal in the absence of sexual desire or stimulation, which can last for hours or even days [3,4]. Symptoms often include genital throbbing, tingling, or a sensation of pressure, typically exacerbated by sitting or stress and relieved only temporarily by orgasm or sleep [5,6]. Although the aetiology of PGAS is largely idiopathic and multifactorial, neurovascular, pharmacological, and psychogenic factors have all been implicated [1,4,5]. In 2009, Waldinger and Schweitzer proposed the concept of restless genital nerves, a syndrome sharing features with restless legs and overactive bladder, suggesting a common neurophysiological mechanism involving sensory hyperexcitability and autonomic dysregulation [5].

Tarlov cysts, also known as perineural cysts, are cerebrospinal fluid-filled dilatations of the nerve root sheaths, most commonly found in the sacral region, particularly at the S2 level [7,8]. These cysts are often incidental findings on MRI, with a reported prevalence of 1.5%-4.6% in lumbosacral imaging studies, though only a minority become symptomatic [8,9].

Emerging evidence suggests that nerve root pathologies, particularly Tarlov cysts, may contribute to PGAS in some patients. While most remain asymptomatic, cysts that compress or irritate adjacent nerve roots can lead to perineal pain, sensory disturbance, and autonomic dysfunction. The pudendal and pelvic splanchnic nerves, which mediate genital sensory and autonomic signalling, arise from the S2-S4 segments [10,11], explaining how lesions at this level may trigger persistent genital arousal.

This report describes a rare case of PGAS secondary to a Tarlov cyst at the S2 level in a 79-year-old woman, highlighting the diagnostic challenges and neurogenic implications of this condition. It contributes to current literature by underscoring an under-recognised presentation of persistent genital arousal associated with Tarlov cysts and emphasises the importance of considering neurological causes in patients with atypical genital sensory symptoms. Neurological features such as lower limb pain, weakness or paraesthesia, bladder or bowel dysfunction, or altered pelvic sensation may provide early clues to underlying neuropathic involvement and should prompt further investigation.

## Case presentation

A 79-year-old woman presented with the sudden onset of increased sexual desire, described as persistent, strong, and unwanted sexual arousal occurring throughout the day and only temporarily relieved by orgasm. The symptoms began shortly after starting estriol vaginal gel, prescribed by her GP for vaginal dryness, discomfort, and burning. Although the onset appeared temporally related to the introduction of vaginal oestrogen, causality could not be established. Her past medical history included heart failure, ischaemic heart disease, frailty, and significant lower limb lymphoedema.

Over the following three months, the symptoms progressively intensified, leading to marked psychological distress, anxiety, and strain on her relationship. She described the sensations as constant and intrusive, interfering with concentration and sleep. Despite multiple consultations with her GP, the symptoms persisted. She was investigated for urinary tract infection and treated for atrophic vaginitis and recurrent candidiasis based on positive swab results. The oestrogen-containing vaginal preparation was subsequently discontinued, but her symptoms continued.

She denied pain, bleeding, or discharge. She was already known to the continence team for incontinence, thought to be secondary to prolapse. There were no systemic symptoms. She reported mild bilateral foot numbness and paraesthesia, first noted in 2022 and previously investigated for peripheral vascular disease. The persistence of these sensory symptoms raised the possibility of underlying neuropathic involvement.

Six months after symptom onset, she was referred to the gynaecology team. Examination revealed a known vaginal prolapse and atrophic vulval and vaginal changes consistent with postmenopausal status, with no other abnormalities. Neurological findings were not documented at that stage. Given the absence of local pathology, a neurological cause, such as involvement of the pelvic or sacral nerve ganglia, was suspected, and a pelvic MRI was requested. A referral was also made for a specialist menopausal opinion, and the GP was advised to refer the patient to neurosurgery.

MRI of the pelvis was declined twice by radiology: initially due to a lack of specific indication and later despite the inclusion of possible Tarlov cysts as a differential. Following discussion at the menopause multidisciplinary team (MDT) meeting, it was recommended to liaise directly with radiology and initiate a trial of low-dose amitriptyline (5-10 mg nocte) or gabapentin for symptomatic relief. A provisional psychosexual referral was also suggested if imaging proved normal.

**Figure 1:**
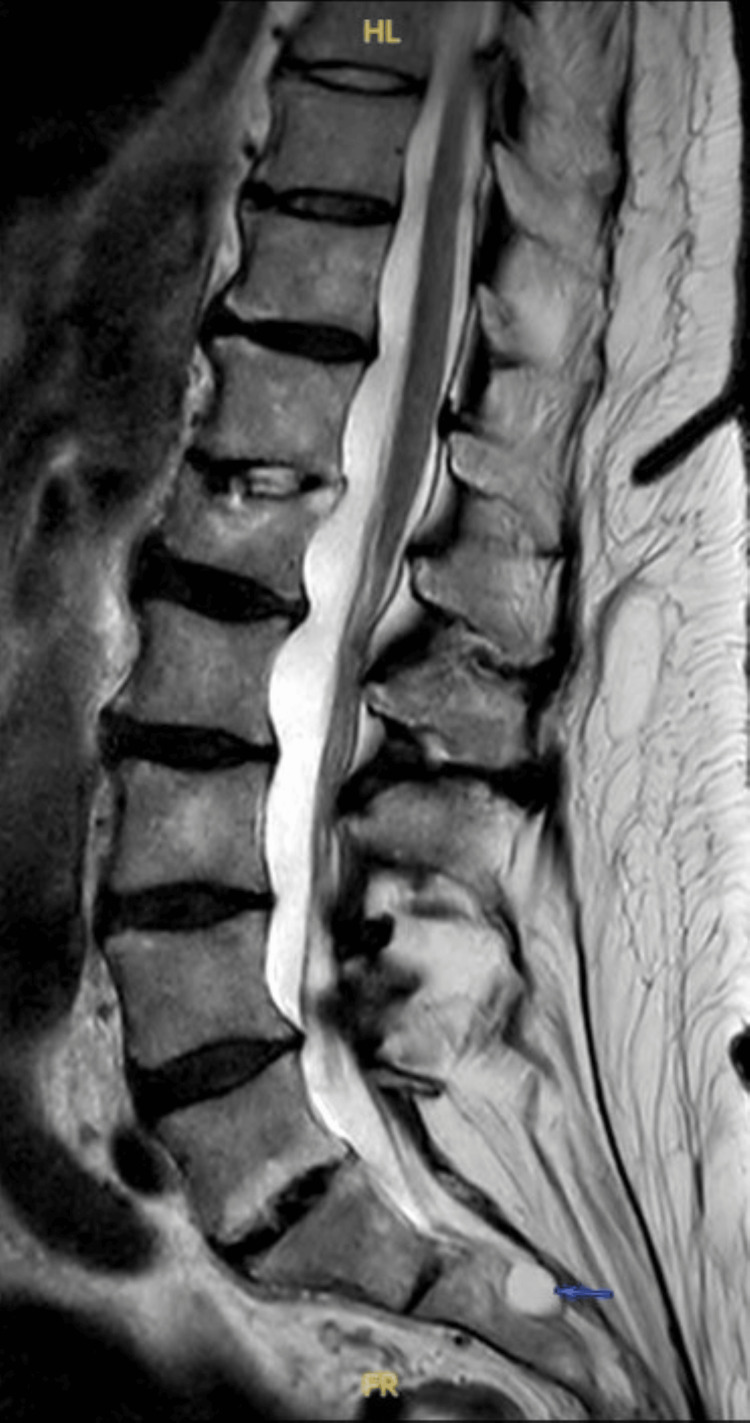
Sagittal T2-weighted MRI showing a sacral Tarlov cyst

Approximately one year after symptom onset, the GP requested an MRI of the lumbar and sacral spine, citing marked kyphoscoliosis, poor straight leg raise, and bilateral lower limb paraesthesia. The MRI demonstrated degenerative spinal changes, a disc bulge at the L4-L5 potentially irritating nerve roots, and small Tarlov cysts at the S2 level and filum terminale (Figure 1). These findings were consistent with possible sacral nerve root irritation contributing to her persistent genital arousal. 

**Figure 2:**
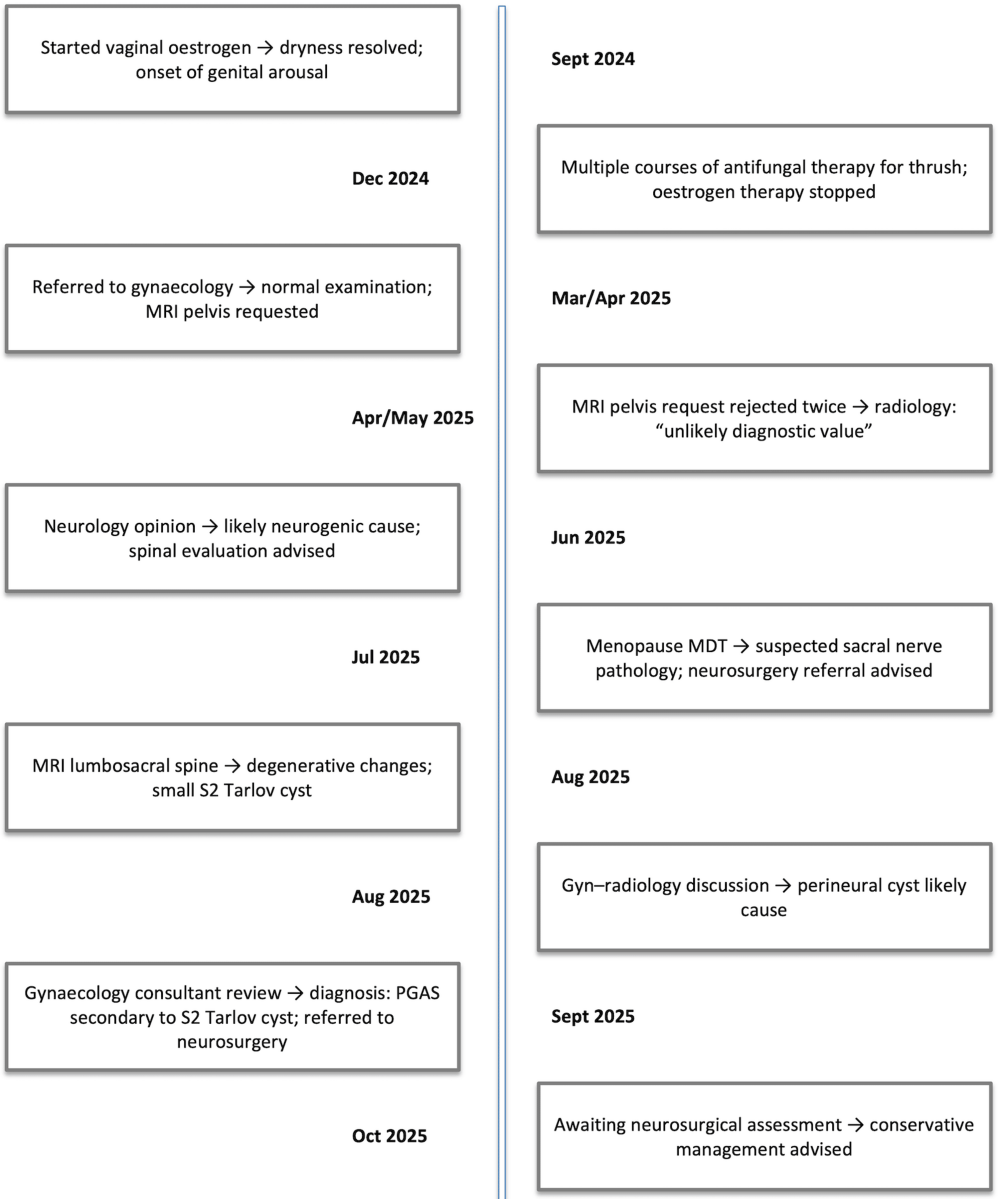
Timeline of clinical events

The patient was subsequently referred to the neurosurgical team for further assessment and ongoing management. At the time of writing, she remains under neurosurgical review. Her symptoms persist and continue to cause distress, although she expressed relief at having a potential neurological explanation. A timeline of clinical events is shown in Figure 2.

## Discussion

The pathophysiology of PGAS is multifactorial (Table 1), involving neurogenic, vascular, pharmacological, endocrine, and psychological components. In recent years, growing evidence has suggested a neurogenic aetiology, particularly implicating the sacral nerves [1]. Among these, Tarlov cysts, arising at the junction of the dorsal root ganglion and the posterior nerve root, have emerged as a significant structural cause in selected cases.

**Table 1:** Pathophysiology of persistent genital arousal syndrome (PGAS)

Proposed mechanism	Pathophysiological basis
Neurovascular compression	Compression or irritation of the sacral nerve roots (S2-S4), particularly via Tarlov cysts, disc bulges, or perineural lesions, may produce sensory hyperexcitability of the pudendal and pelvic splanchnic nerves, resulting in spontaneous arousal and paraesthesia [7-11]
Peripheral sensory hyperexcitability/"restless genital nerves"	Abnormal firing of genital afferents analogous to restless legs syndrome (RLS), causing spontaneous genital sensations independent of sexual stimuli; may coexist with overactive bladder or RLS symptoms [5,12]
Small-fibre neuropathy/altered sensory signalling	Dysfunction of small unmyelinated C-fibres in genital sensory nerves or dorsal root ganglia, leading to abnormal genital sensation, tingling, or throbbing even without stimulation [1,2]
Serotonergic and dopaminergic dysregulation	Antidepressant use or withdrawal (especially SSRIs/SNRIs) may disrupt serotonin-dopamine balance in sexual arousal pathways, triggering or exacerbating PGAD symptoms [3,4]
Autonomic dysregulation	Abnormal sympathetic-parasympathetic interplay within pelvic autonomous plexus leading to prolonged genital vasocongestion and heightened arousal without sexual desire [1,5]
Psychogenic and affective modulation	Psychological distress, hypervigilance, or anxiety may perpetuate or amplify symptoms through cortical and limbic arousal circuits [2,6]
Hormonal/pharmacological influences	Local oestrogen exposure, antidepressant initiation or cessation, and neuroactive medication effects may alter genital sensory thresholds [4,6]
Multifactorial/biopsychosocial model	Integration of neurogenic, vascular, hormonal, and psychological mechanisms best explains the heterogeneity of PGAS presentations and emphasises multidisciplinary evaluation [2,11]

Tarlov cysts, which occur in approximately 4% of the population, are usually asymptomatic [7]. However, when located near the S2-S4 nerve roots, they may irritate these roots, leading to sensory, motor, or autonomic symptoms. The pudendal nerve, which arises from these roots, mediates genital sensation and arousal, explaining the association between cyst location and PGAS manifestation [10,11].

In this case, the close anatomical relationship between the S2 Tarlov cyst and the genital sensory pathways, together with a normal pelvic examination and the absence of psychiatric or pharmacological triggers, strongly supports a neurogenic aetiology. The concurrent bilateral lower limb paraesthesia further reinforces this, suggesting broader lumbosacral involvement rather than isolated pudendal dysfunction. These sensory changes likely reflect partial nerve root irritation arising from both the Tarlov cyst and underlying degenerative spinal disease. The temporal overlap with vaginal oestrogen use is most plausibly coincidental, as no evidence supports a causal link between hormonal therapy and persistent autonomic arousal.

The diagnostic process was complex, involving gynaecological, neurological, and menopausal specialists before a sacral cyst was suspected and eventually confirmed. This multidisciplinary course underscores the diagnostic challenges of PGAS, where symptoms are often misattributed to psychosexual or endocrine causes before imaging is pursued. Early recognition of subtle neurological symptoms, such as paraesthesia or altered perineal sensation, may help clinicians consider a neurogenic origin sooner, reducing diagnostic delay and improving quality of life. A limitation of this case is that no axial MRI slices were available at the level of the cyst, and a pelvic MRI was not performed, restricting detailed anatomical assessment of the sacral nerve roots. MRI remains the gold standard for diagnosing Tarlov cysts and should be considered in patients presenting with unexplained genital arousal or perineal sensory changes.

Treatment of PGAS secondary to Tarlov cysts depends on symptom severity and cyst size. Conservative management, including neuropathic pain medication, pelvic floor physiotherapy, and psychosexual support, may benefit some patients [6]. In other symptomatic cases with clear radiological correlation, surgical options such as cyst fenestration, fibrin-glue injection, or microsurgical excision have produced partial or complete symptom relief in case series [8,9], though outcomes remain variable. Given this patient’s advanced age, frailty, multiple comorbidities, and the small size of the S2 cyst, a conservative and multidisciplinary management plan was deemed most appropriate. Initial treatment focused on symptomatic relief through neuropathic pain modulation and psychosexual support rather than invasive intervention. Ongoing follow-up through combined gynaecology and neurosurgery services will ensure that, if conservative measures prove ineffective, escalation to targeted or interventional treatments can be considered in a controlled, evidence-based manner.

## Conclusions

This case report highlights a rare neurogenic cause of PGAS associated with an S2 Tarlov cyst. It underscores the importance of considering underlying lumbosacral pathologies when standard gynaecological and urological investigations are unremarkable. Diagnostic delays in PGAS are common due to its atypical presentation, limited clinical awareness, and the sensitive nature of symptoms, factors that can lead to significant psychological and relational distress for patients. An individually tailored, multidisciplinary approach is essential to balance the benefits and risks of treatment in the context of patient comorbidities and other factors. Greater awareness of neurogenic mechanisms and earlier recognition of atypical presentations may help reduce diagnostic delays, facilitate timely treatment, and ultimately improve patient outcomes.

## References

[1] Jackowich RA, Pink L, Gordon A, Pukall CF (2016). Persistent genital arousal disorder: a review of its conceptualizations, potential origins, impact, and treatment. Sex Med Rev.

[2] Jackowich RA, Pukall CF (2020). Persistent genital arousal disorder: a biopsychosocial framework. Curr Sex Health Rep.

[3] Leiblum S, Brown C, Wan J, Rawlinson L (2005). Persistent sexual arousal syndrome: a descriptive study. J Sex Med.

[4] Leiblum SR, Goldmeier D (2008). Persistent genital arousal disorder in women: case reports of association with anti-depressant usage and withdrawal. J Sex Marital Ther.

[5] Waldinger MD, Schweitzer DH (2009). Persistent genital arousal disorder in 18 Dutch women: part II - a syndrome clustered with restless legs and overactive bladder. J Sex Med.

[6] Rosenbaum TY (2010). Physical therapy treatment of persistent genital arousal disorder during pregnancy: a case report. J Sex Med.

[7] Lucantoni C, Than KD, Wang AC, Valdivia-Valdivia JM, Maher CO, La Marca F, Park P (2011). Tarlov cysts: a controversial lesion of the sacral spine. Neurosurg Focus.

[8] Murphy K, Oaklander AL, Elias G, Kathuria S, Long DM (2016). Treatment of 213 patients with symptomatic Tarlov cysts by CT-guided percutaneous injection of fibrin sealant. AJNR Am J Neuroradiol.

[9] Abdi D, Huttunen J, Leinonen V, Savolainen S, Danner N (2023). Operative treatment of Tarlov cysts - outcomes and predictors of improvement after surgery: a series of 97 consecutive patients and a systematic review of literature. Global Spine J.

[10] Lim VM, Khanna R, Kalinkin O, Castellanos ME, Hibner M (2020). Evaluating the discordant relationship between Tarlov cysts and symptoms of pudendal neuralgia. Am J Obstet Gynecol.

[11] Murphy K, Nasralla M, Pron G, Almohaimede K, Schievink W (2024). Management of Tarlov cysts: an uncommon but potentially serious spinal column disease-review of the literature and experience with over 1000 referrals. Neuroradiology.

[12] Waldinger MD, Venema PL, van Gils AP, Schutter EM, Schweitzer DH (2010). Restless genital syndrome before and after clitoridectomy for spontaneous orgasms: a case report. J Sex Med.

